# The analysis of the implementation of RBL-STEM learning materials in improving student’s meta-literacy ability to solve wallpaper decoration problems using local antimagic graph coloring techniques

**DOI:** 10.1016/j.heliyon.2023.e17433

**Published:** 2023-06-19

**Authors:** Dafik, Kadir, T.K. Maryati, Sufirman, Z.R. Ridlo

**Affiliations:** a Department of Mathematics Education Postgraduate, Universitas Jember, Indonesia; b Department of Mathematics Education, UIN Syarif Hidayatullah Jakarta, Indonesia; c Department of Mathematics Education, Universitas Jember, Indonesia; d Department of Sciences Education, Universitas Jember, Indonesia; e PUI-PT Combinatorics and Graph, CGANT, Universitas Jember, Indonesia

**Keywords:** Local antimagic graph coloring, Meta-literacy, RBL-STEM, Tessellation wallpaper

## Abstract

The tessellation problem is interesting to study, especially when it is associated with mathematical concepts. In this study, a graph coloring technique will be applied to solve the problem of wallpaper tessellation decoration. The main objective of this study is to improve students' meta-literacy abilities when applying coloring techniques in completing tessellation wallpaper decorations in RBL-STEM learning. RBL is a learning model that stands for Research-Based Learning. This model is now becoming the focus of attention of learning practitioners, while the STEM approach is an approach that involves four studies in the field of science, namely science, technology, engineering, and mathematics. The method used in this study is a mixed method, a combination of quantitative and qualitative. Quantitative methods were used to analyze the significant differences in the students' meta-literacy learning achievement between the control and experimental classes. In contrast, the qualitative method was used to analyze the results of in-depth interviews, a triangulation process from the quantitative research results. The results of this study indicate that there is a significant difference in meta-literacy ability between the control class (the class that applies RBL-STEM but without using the learning materials developed by the researcher) and the experimental class (the class that applies RBL-STEM using the learning materials developed by the researcher). The independent sample *t*-test on the post-test learning outcomes of meta-literacy abilities on Sig (2 Tailed) showed a significant difference of 0.013 < 0.05. Furthermore, the data on the meta-literacy ability of students showed that as much as 10% of students have the poor meta-literacy ability, 17% of students have fair meta-literacy ability, 26% of students have the good meta-literacy ability, 32% of students have very good meta-literacy ability and 15% of students have the excellent meta-literacy ability. Based on this research result, to improve the students' meta-literacy, we need to implement the learning method which fosters the research activity in the classroom and brings the real-life phenomenon in the classroom. The integration of RBL and STEM is one of the novel breakthroughs.

## 1 Introduction

Meta-literacy is the literacy that promotes critical thinking and collaboration in the digital age, providing a comprehensive framework for participating effectively in social media and online communities. Meta-literacy is an integrated construct that supports the acquisition, production, and sharing of knowledge in collaborative online communities and simultaneously integrates information literacy with other types of literacy. Meta-literacy is needed since information literacy is not enough to adapt to the development of social technology [1]. Meta-literacy is an ability that goes beyond metacognition and technological literacy. See the indicators and sub-indicators of meta-literacy in Table 1.

**Table 1:** Meta-literacy indicators and sub-indicators.

Indicators	Sub-indicators
Produce	P1: Identify the nature or characteristic of the problem
	P2: Develop breakthroughs for problem-solving
	P3: Define the stages, phases, syntax, or algorithms
Incorporate	I1: Identifying patterns of solutions
	I2: Generalizing the solution
	I3: Using the IoT, such as platforms or applications, to integrate results
Use	U1: Test or assess the results
	U2: Analyzing the obtained results
	U3: Perform interpretation and prediction
	U4: Utilize the results
Share	S1: Use IoT (Social Media, OER, MOOCs, Teaching Platform)
	S2: Distribute the results followed by reflection and evaluation
	S3: Evaluation of the feedback
	S4: Forecasting the response trend with software application
Collaborate	C1: Working together using the Internet of Things technology
	C2: Ask for suggestions from others
	C3: Encourage people to do more to contribute findings
	C4: Obtaining joint works for publication
	C5: Determining future work together for the wider community

However, in general, in Indonesia, students' meta-literacy abilities are still relatively low. One of the causes of the common ability is that the learning model that has been applied in the classroom does not support the rise of this ability. The research to propose a meta-literacy model to redefine information literacy, but the result did not show what specific model that can be used to raise the student's meta-literacy [1]. The study on enhancing students' blended learning experience through embedding meta-literacy. This study also did not show the significant influence of blended learning on improving meta-literacy [2]. The study which focused on determining the pedagogy method of care for information literacy and meta-literacy asynchronous online instruction [3]. He suggests some cooperative learning models that can be incorporated to promote the student's meta-literacy. Thus, some educators have been implementing cooperative learning models, but they do not really focus on engaging students to the use of Internet of Things in the classroom, such as software applications, software programming, a teaching platform, a crowdsourcing platform, and massive online open courseware. Thus, there is a big gap between the student's learning outcome and the development of disruptive technology currently.

Therefore, in this study, an RBL learning model integrated with the STEM (Science, Technology, Engineering, Mathematics) approach will be applied to solve the problem of wallpaper decoration using local antimagic graph coloring techniques [4].

The application of Research-Based Learning (RBL) refers to the findings published by several papers. The case studies focused on professional learning and development in bachelor's excellence program of Maastricht University, Netherlands. The authors introduced new forms of problem-based learning in the format of research-based learning [5]. The research investigating the effectiveness of Research-Based Learning (RBL) related to students' learning achievement in solving two-dimensional arithmetic sequence problems was carried out in Ref. [6]. It showed that the research-based learning model contributes to the students such that they are more active and creative, and students think more critically. This is due to that RBL provides a research atmosphere for students such that they are trained to have creative and innovative thinking skills [6]. The implementation of RBL model integrated with computer programming has also a significant impact to higher thinking skills, since the two combinations effectively speed up the existence of students' analytical process under the systematics research activities [7]. Another result related to implementation of RBL model in improving the student's metacognition skills was studied in Ref. [8] specifically in solving division operation problems.

In regards with STEM, there are also some results published in some journals. The STEM learning activities in making an alternative natural preservative for processed meat from shrimp skin, namely chitosan, together with RBL models, RBL-STEM effectively motivates the student get the systematics steps in making the product [9]. The STEM approach also effective in solving graph rainbow antimagic coloring problems since this approach helps the student to improve their meta-literacy [10]. The research about the development of RBL-STEM learning materials to improve students' combinatorial thinking skills in solving local (a, d)-edge antimagic coloring problems was also studied in Ref. [11]. The research of STEM approach focused to design a parachute is also effective in improving the students’ combinatorial thinking skills [12]. The last is the study of STEM approach combined with lesson study, it also showed that the combination of Lesson Study and STEM gained an effective learning cycle [13].

Related to the local antimagic graph coloring, we give the definition as follows: Let *G* = *(V,E)* be a connected graph with *|V|* = *n* and *|E|* = *m*, A bijection *f: E → {1,2, …, m}* is called local antimagic labeling if for any two adjacent vertices *u* and *v*, *w(u)* ≠ *w(v)* where *v* is the set of edges incident to *u*. Thus any local antimagic labeling induces a proper vertex coloring of *G* where the vertex *v* is assigned the color *w(v)*. The local antimagic chromatic number, denoted by *χ*_*la*_*(G),* is the minimum number of colors taken over all colorings induced by local antimagic labelings of *G. S*ome relevant results about coloring of prism graph and its central, middle, total and line graph [14]. The study about local irregular vertex coloring of comb product by path graph and star graph [15]. There are also some studies on irregular coloring of triple star graph families, see Ref. [16], the conjecturing process: perspectives in theory and implementation, see Ref. [17] and the study of local antimagic vertex coloring of graph, see Ref. [18].

**Fig. 1:**
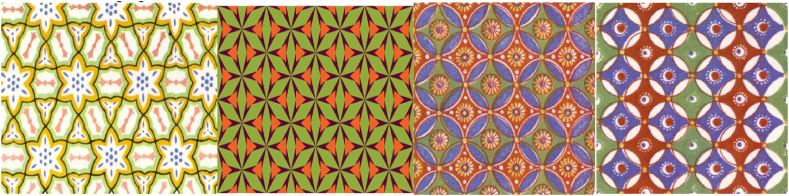
Illustration of a wall wallpaper tessellation decoration.

Tessellation itself is a process on closing one or more gaps by using geometric shapes. The application of tessellation is very widely used. For example, the tessellation of paving blocks and tessellation of wallpaper, including batik motifs, can also use the concept of tessellation. To have an excellent wallpaper tessellation decoration, the wallpaper must be designed with symmetrical and good coloring patterns. In this problem, students develop the wallpaper decoration in the form of local edge antimagic coloring to find the appropriate pattern and color combination. Furthermore, by using local antimagic vertex coloring of graphs, students can discover symmetrical designs and good coloring patterns. It is the novelty of this research, since to have wallpaper decoration so far, they just use a color combination by means of software application. Fig. 1 shows an example of tessellation decoration of the wallpaper.

The main objective of this research is to solve the following problems: (1) How is the process of the implementation of RBL-STEM approach improving students' meta-literacy ability on solving wallpaper decoration using local antimagic graph coloring techniques? (2) Is the effectiveness implementation of the RBL-STEM effective to improve students' meta-literacy ability on solving wallpaper decoration using local antimagic graph coloring techniques?

To solve the above problems, we use the following strategies: (1) Utilize the learning materials of RBL-STEM which have developed by authors to analyze the process of the implementation of RBL-STEM approach in improving students' meta-literacy ability on solving wallpaper decoration using local antimagic graph coloring techniques; (2) We give a specific task to the students, namely obtaining the local antimagic coloring of the graph. Further, under the implementation of the RBL-STEM approach, we encourage the students to use the obtained local antimagic coloring of the graph to solve the wallpaper decoration problem, and then we analyze the significant improvement of the students' meta-literacy ability.

**Fig. 2:**
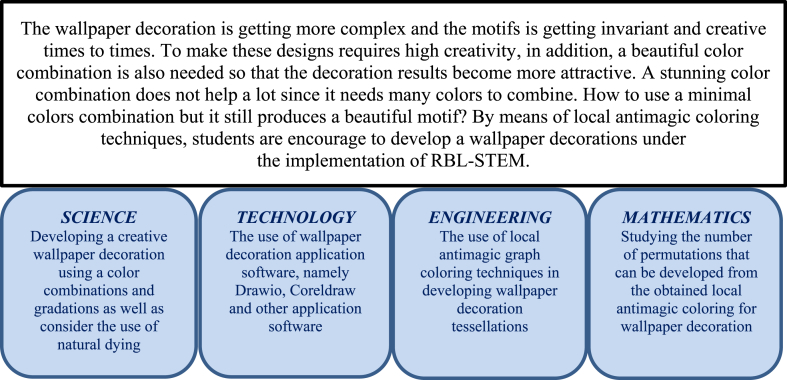
STEM problems in wallpaper tessellation decoration problems.

Fig. 2 shows the four elements of STEM approach dealing with the implementation of the RBL-STEM in improving the students' meta-literacy ability on solving wallpaper decoration using local antimagic graph coloring techniques.

The complete student's activity in STEM approach consists of four aspects, namely Science, Technology, Engineering and Mathematics. In science activities, students determine the wallpaper tessellation decoration as the basic construction, and also understand the characteristics of the wallpaper. In technology activities, students use of wallpaper decoration application software, namely Drawio, Coreldraw and other application software. In engineering activities, students utilize the local antimagic graph coloring techniques in developing wallpaper decoration tessellations. In mathematics activities, students include the basic construction of wallpaper tessellation to construct a wider area wallpaper and obtain the permutation of pattern to obtain as many as different pattern for having a different wallpaper tessellation motifs.

## 2 Research methods

### 2.1 Approaches and types of research

**Fig. 3:**
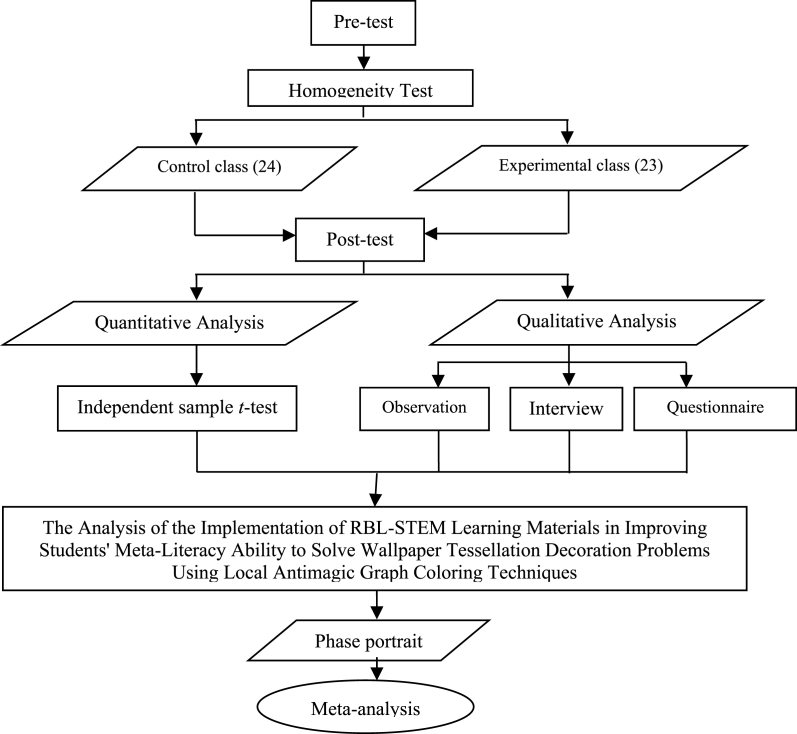
Flowchart of experimental design.

The method used in this study is a mixed method, namely the combination of quantitative and qualitative methods. The quantitative method is used to analyze the significant differences in students' meta-literacy between the control and experimental classes, see Table 2. In contrast, the qualitative method is used to analyze the portrait phase of students' meta-literacy under the implementation of RBL-STEM [19]. The qualitative method was also used to analyze the results of in-depth interviews as the triangulation process against the quantitative research results [20]. Globally the research design can be illustrated in Fig. 3.

**Table 2:** Quasi-experiment research design.

Class	Pre-test	Treatment	Post-test
Experiment class	O_1_	X	O_2_
Control class	O_3_	–	O_4_

Notes: O_1_, O_3_: Pre-test given to both class groups to see the initial condition of students' meta-literacy abilities. X: Implementation of the RBL-STEM learning materials using local antimagic graph coloring in solving the problem of wallpaper tessellation decoration. O_2_, O_4_: Post-test given to both class groups to see the final condition of students' meta-literacy abilities after RBL-STEM implementation.

### 2.2 Data collection procedure

The data collection procedure complied with research ethics standards and was approved by the Research Ethics Committee of the University of Jember, Indonesia. The data collection steps was carried out in the following stages: 1) The researcher as well as lecturer prepared the syllabus, lesson plans, students worksheet and assessment instruments that had been validated by the expert validators and then brought into the lecture class to carry out the learning cycle in Discrete Mathematics course, 2) The researchers gave a pre-test to have the prior students' meta-literacy ability and we categorized the results into five categories, namely poor, fair, good, very good and excellent, 3) The researcher carried out the learning cycles on the local edge antimagic coloring by mean of student worksheets and then observed the students learning activities using the observation sheet instrument, 4) On each learning cycle, students applied the obtained local edge antimagic coloring of graph by means of student worksheets in solving wallpaper decoration, 5) At the final stage of the learning cycle, students were given a post-test to analyze their meta-literacy ability, 6) The assessment was carried out through a post-test of students' meta-literacy ability and we categorized the results into five categories too, namely poor, fair, good, very good and excellent, 7) In the last stage, we distributed the questionnaire to all respondents to have the capture of their meta-literacy ability as secondary data and conducted some interviews to the selected respondents for the purpose of visualizing the portraits phase of the student's meta-literacy ability for triangulation stage.

### 2.3 Participants

The population of the research is undergraduate students of the University of Jember, Jember, East Java, Indonesia, who joint the Discrete Mathematics course in the 2022 academic year. The selection of this population was permitted by the Research Ethics Committee and the Dean of the Faculty of Teacher Training and Education. The sample selection uses a proportional random sampling for determining the control class (as many as 24 students), and the experimental class (as many as 23 students) respected to the research proposal, which has been reviewed by an independent ethic commission, namely SREC (Social Research Ethic Committee), UHAMKA Indonesia of number: 142/F.03.01/2022.

### 2.4 Instruments

The instruments in this study consist of the syllabus, RPS, LKM, LHM (Pre-test and Post-test of students' meta-literacy abilities), student activity observation sheets, interviews, and questionnaires [21]. The scale used is a Likert scale with an interval of 1–4. All instruments have passed the validation test stage by experts published in a separate paper from this study [22].

### 2.5 Data analyses procedure

There are five steps of the data analyses procedure, namely (1) Validity and reliability test by using Pearson correlation and Cronbach's Alpha statistic, (2) Homogeneity test of two respondent classes by using Levene statistic, (3) Normality test of two respondent classes using Kolmogorov-Smirnov normality and Shapiro-Wilk tests, (4) Mean difference analysis using student *t*-test to test whether there is a significant difference of students' meta-literacy between the experimental class and the control class under the implementation of RBL-STEM, lastly (5) RRA (Real Relative Asymmetry) analysis to test the integrity on the node of students meta-literacy phase portrait.

### 2.6 Research hypothesis

There are two hypotheses proposed in this study. Those are as follows.

• *H*_*0*_: There is no difference in students' meta-literacy abilities between the control class and the experimental class in solving the problem of wallpaper decoration using local antimagic graph coloring techniques under the implementation of RBL-STEM learning materials.
• *H*_*1*_: There is a significant difference in students' meta-literacy abilities between the control class and the experimental class in solving the problem of wallpaper decoration using local antimagic graph coloring techniques under the implementation of RBL-STEM learning materials

The hypothesis is written as a pair of the null hypothesis (*H*_0_) and alternative hypothesis (*H*_1_). To consider the acceptance of the hypothesis, the following rules should be satisfied, namely, if the value of Sig. (2 Tailed) > 0.05, then *H*_0_ is accepted, and *H*_1_ is rejected, but if the value of Sig. (2 Tailed) < 0.05, then *H*_0_ is rejected, and *H*_1_ is accepted.

## 3 Research findings

### 3.1 RBL-STEM implementation stages

**Fig. 4:**
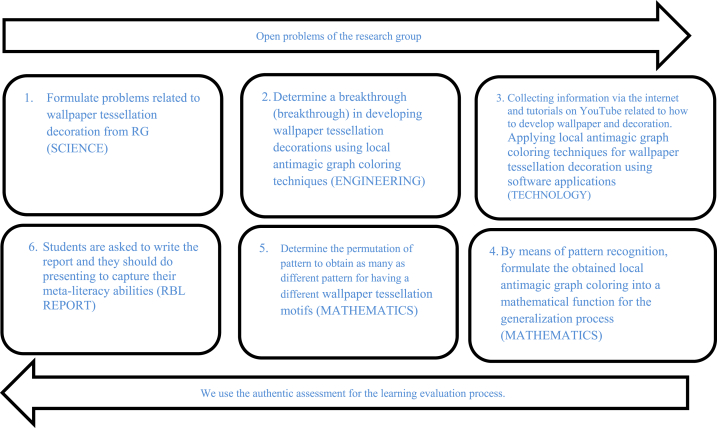
The syntax of the RBL model with STEM approach in wallpaper tessellation decoration using local antimagic graph coloring technique.

In the following, we will show the research results on solving the problems described in the introduction. The answer to the first problem formulation is implementing the RBL model in the STEM approach to improve students' meta-literacy abilities to solve the problem of wallpaper decoration using local antimagic graph coloring techniques. The integration of the RBL model with the STEM approach is applied using the syntax in Fig. 4.

**Fig. 5:**
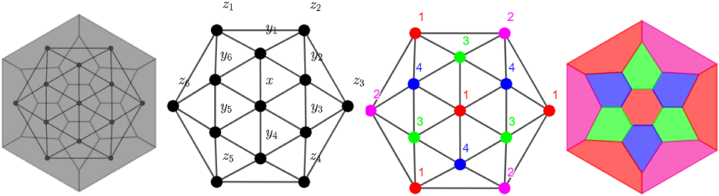
The Illustration of the development of tessellation constructions using local antimagic graph coloring techniques.

**Fig. 6:**
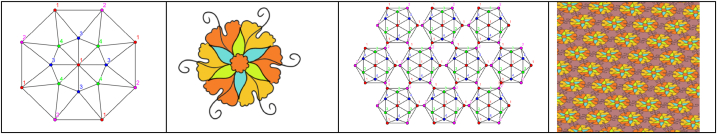
The Illustration of the development of wallpaper decorations using local antimagic graph coloring techniques.

The students were given the STEM problems, namely: 1) Determining the wallpaper tessellation as the basis for construction and understanding the characteristics of the wallpaper (Science), 2) Representing it in graph form with the help of software (Technology), 3) Obtaining the local antimagic graph coloring (Engineering), see one of the results of the coloring technique in Fig. 5, then 4) Generalizing or expanding the obtained color and applying it for wallpaper tessellation decoration (Engineering), see the results in Fig. 6, next 5) Using the basic construction of tessellation to construct wallpaper with a wider area (Mathematics), (6) Determining the permutation of pattern to obtain as many as the different pattern for having a different wallpaper tessellation motifs (Mathematics).

### 3.2 The analysis of the implementation of the RBL-STEM model

This section will describe whether the implementation of RBL-STEM can improve students' meta-literacy abilities in solving the problem of wallpaper decoration using local antimagic graph coloring techniques or not. By utilizing the existing learning materials developed, we implemented those learning materials, which consist of the syllabus, RPS, LKM, LHM (Pre-test and Post-test of students' meta-literacy abilities), observation sheets, interviews sheets, and questionnaires sheets. All of the above materials have been validated, respected to the content validity, format validity, language validity, and practicality and the effectiveness of the use of RBL-STEM learning materials.

**Fig. 7:**
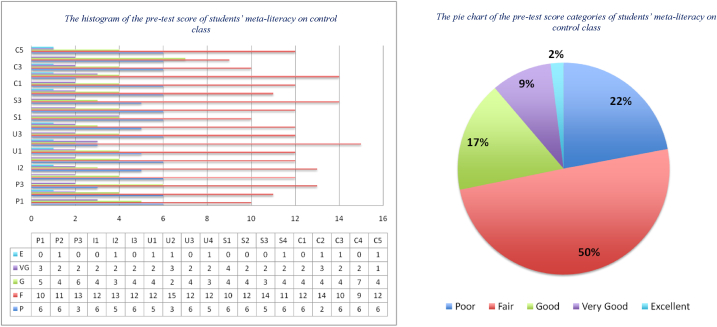
The distribution of the pre-test score of students' meta-literacy in the control class.

**Fig. 8:**
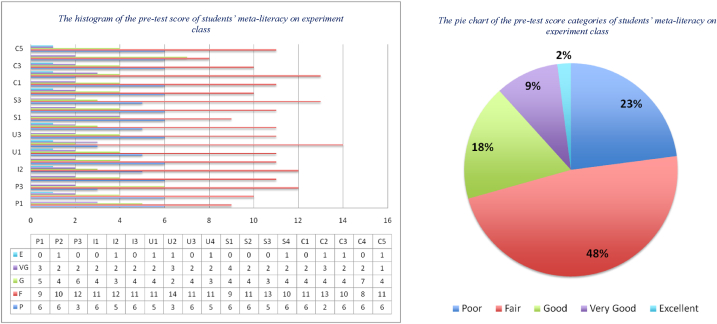
The distribution of the pre-test score of students' meta-literacy in experiment class.

An important step in implementing these learning materials is conducting a pre-test of students' meta-literacy abilities, and the results can be depicted in Fig. 7, Fig. 8. Another important thing is to test the validity and reliability of the pre-test instrument on students' meta-literacy ability. The test instrument is derived from the main indicators and sub-indicators of the meta-literacy. The number of pre-test questions is as many as the number of meta-literacy sub-indicators, i.e., 19 questions. By using Pearson Correlation inferential statistics, the results of the validity test obtained that the value of Sig. (2-tailed) of almost all question items are less than 0.05, and only 1 question is above 0.05. Therefore, we replaced it with another equivalent question and then retested it until the value of Sig. (2-tailed) < 0.05. We also tested Cronbach's Alpha score to show the reliability test, and it gave a score of 0.793, which is greater than 0.60, see Table 3. Thus, it can be concluded that the pre-test instrument is reliable.

**Table 3:** The reliability test of the pre-test instrument.

Reliability statistics		
Cronbach's alpha	Cronbach's alpha, based on standardized items	N of items
0.799	0.787	19

Furthermore, we also tested the homogeneity of two classes of respondents to know the similarity of those classes. The results of the quasi-experimental homogeneity test can be seen in Table 4.

**Table 4:** Test of homogeneity of variances.

Value			
Levene statistic	df1	df2	Sig.
0.095	1	48	0.786

Based on the results of the homogeneity of variance test in Table 4, it is known that the significance value (Sig.) of the students' meta-literacy ability pre-test is 0.691. Since the value of Sig. 0.786 > 0.05, it can be concluded that the variance of the pre-test of meta-literacy ability of control and experimental classes is homogeneous.

**Fig. 9:**
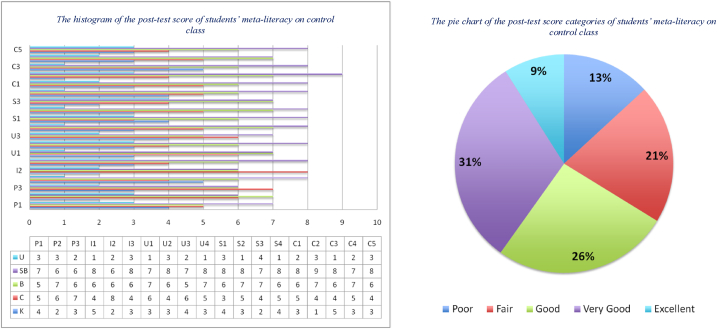
The distribution of the post-test score of students' meta-literacy in the control class.

**Fig. 10:**
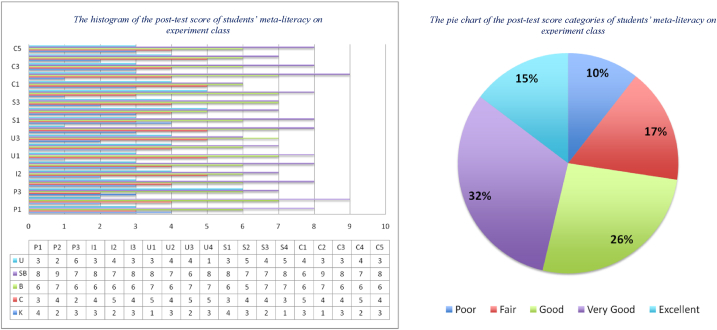
The distribution of the post-test score of students' meta-literacy in experiment class.

The last step is to test the difference between the two means of the post-test derived from the control and experimental classes. This aims to determine the effect of the implementation of RBL-STEM learning materials in improving students' meta-literacy ability to solve wallpaper decoration problems using local antimagic graph coloring techniques. From the research design in Table 2, it is known that in the experimental class, RBL-STEM learning was applied together with learning materials developed by researchers. At the same time, the control class was also applied to RBL-STEM learning but did not use learning materials developed by researchers. The post-test results can be seen in Fig. 9, Fig. 10.

Prior to analyzing the post-test scores using the independent sample *t*-test, we did a normality test on the post-test score of students' meta-literacy abilities. The results of the normality test can be seen in Table 5.

**Table 5:** Tests of normality.

	Kolmogorov-Smirnov^a^			Shapiro-Wilk		
	Statistic	df	Sig.	Statistic	df	Sig.
Value	0.142	49	0.213	.867	49	0.428

a Lilliefors Significance Correction.

The significance value of the Kolmogorov-Smirnov test is 0.213 (p > 0.05). Thus, based on the Kolmogorov-Smirnov normality test, the post-test value data is normally distributed. The significance value in the Shapiro-Wilk test is 0.428 (p > 0.05), so the data is also normally distributed based on the Shapiro-Wilk normality test.

Finally, a statistical test will be carried out using an independent sample *t*-test to test whether there is a significant difference of students' meta-literacy between the experimental class and the control class under the implementation of RBL-STEM in solving the problem of wallpaper tessellation decoration learning problem using local antimagic graph coloring technique. Table 6 shows the results of the *t*-test at a confidence level of 5% Sig value. (2-tailed) = 0.002 < 0.05. This means that *t*_*count*_ *> t*_*table*_, which concludes *H*_*0*_ is rejected and *H*_*1*_ is accepted. Thus, it can be stated that there is a significant difference in meta-literacy ability between the control and experimental classes under the implementation of the RBL-STEM in the Discrete Mathematics class. The conclusion is that the implementation of the RBL-STEM learning material can improve the meta-literacy ability of students to solve the problem of wallpaper tessellation decoration using local antimagic graph coloring techniques.

**Table 6:** Independent samples *t*-test.

		Levene's test for equality of variances		*t-*test for equality of means						
		F	Sig.	T	df	Sig. (2-tailed)	Mean difference	Std. error difference	95% confidence interval of the difference	
									Lower	Upper
Score	Equal variance assumed	0.092	0.772	−2.806	49	0.002	−2.233	0.764	−3.831	−0.643
	Equal variance not assumed			−2.819	46.59	0.002	−2.233	0.765	−3.832	−0.632

To substantiate the above statistical results, Table 7 illustrates the comparison of descriptive data of the control class and the experimental class. From Table 7, it is clear that the maximum, average, and standard deviation of the experimental class showed better data than the control class. In conclusion, the meta-literacy ability of students in the experimental class is better than that in the control class in solving the problem of wallpaper tessellation decoration using the local antimagic coloring graph technique.

**Table 7:** The descriptive data of the student's meta-literacy under the implementation of RBL-STEM.

	N	Min	Max	Mean	Std. deviation
Pre-test of control class	24	56	70	61.50	.815
Post-test of control class	24	62	79	72.00	.942
Pre-test of experiment class	23	68	73	70.00	1.118
Post-test of experiment class	23	73	89	83.50	1.253

### 3.3 Phase portrait analysis of the student meta-literacy ability

**Fig. 11:**
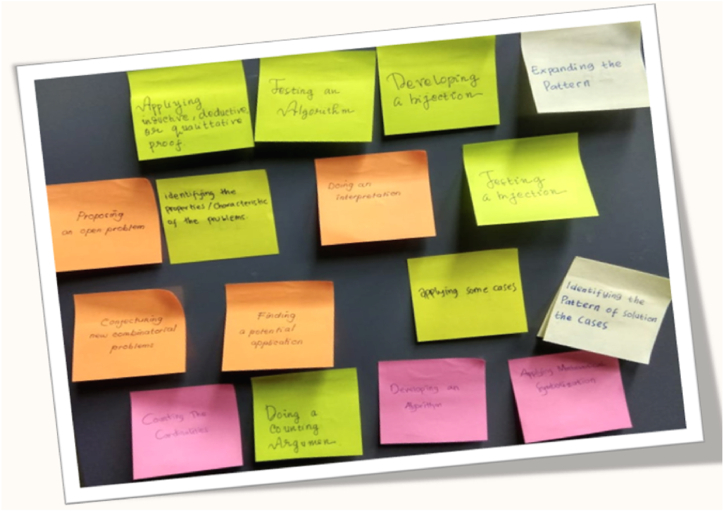
The phase portrait interview cards to obtain a phase portrait of the student's meta-literacy ability of the research subject.

Phase portraits of student meta-literacy abilities are schematic visualizations obtained from the process of acquiring literacy abilities through interviews with the students of the experiment class by using phase portrait interview cards. A phase portrait interview card is a card that contains sub-indicators used by researchers to conduct in-depth interviews of research subjects. There are 19 sub-indicators of meta-literacy ability, so the number of cards is 19, see Fig. 11. The inscription in each card is derived from the meta-literacy sub-indicators, and then behind the cards are given a code according to the codification of the meta-literacy sub-indicators such as P1, P2, P3, I1, I2, … and so on. At the time of the interview process, the interviewee did not understand the existence of these card codes. While posing the problem taken from the post-test questions, the researcher conducted an interview to ask the student's thinking process on solving the problem of wallpaper tessellation decoration using the local antimagic coloring graph technique. Each answer is matched with its code then its connection is embodied in a phase portrait image.

The card was given to the selected research subject of the meta-literacy ability criteria, namely excellent, very good, good, fair, and poor. Transcripts of interviews are recorded, then adjusted to the phase portrait interview cards, recorded the code, and matched with its code. Then, its connection is embodied in a phase portrait graph. In this study, only three-phase portraits will be shown, namely phase portraits of student meta-literacy ability from the experimental class with the categories Excellent (S20KE), Good (S12KE), and Poor (S8KE). Here is an example of an interview transcript with the S20KE research subject.

**Table:** 

Interview with S20KE		
R	:	If you encounter a problem with the tessellation decoration of the wallpaper which you see in the worksheet, what is the first time you imagine?
S20KE	:	Consider the beauty of the decoration and determine how to develop it
R	:	You have been taught how to develop it with local antimagic graph coloring techniques, can you properly develop it?
S20KE	:	Right sir, I have already understood and tried to develop it but sometimes it is a bit difficult to determine the pattern?
R	:	Well, try to pay attention to these phase portrait interview cards, it is containing the stages to solve the problem of wallpaper tessellation decoration, try to start with P1 and think about what is the next step do you decide?
S20KE	:	Well sir, I will try to determine what is the pattern of this decoration and I will find the strategy using local antimagic coloring of graph
R	:	Good, that's a step related to P2. Your technique is unique and can be used as a breakthrough to solve the problem, and please continue what else are you going to do?
S20KE	:	Well, sir, I'm trying to guess roughly what is the pattern for a larger graph?
R	:	Yes, please go ahead it is already in step I1, and there is no problem. Please try and continue. What other steps are you going to develop?
S20KE	:	Yes, sir I have found a pattern, this I will check for a larger graph whether it is still applicable this pattern to other larger order of graph.
R	:	Good, please keep going, you've been in stage I2, do in the same way and then think after this which card will you choose?
S20KE	:	What about this step, sir? Now, I can have some hints for doing the next stages.
R	:	Yes, please do whatever you choose, but the most important thing you can gain the hints.
		… … … … … … … … … … … … … … … … … … … …
		to be continued

**Fig. 12:**
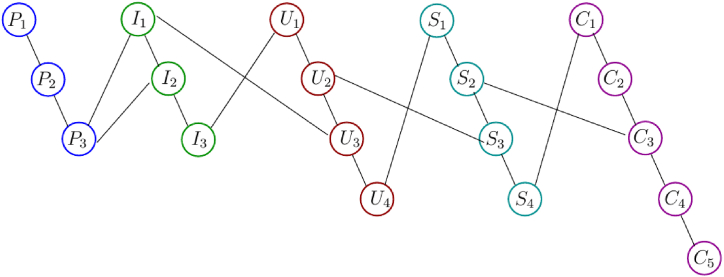
The phase portrait of the meta-literacy ability of student S20KE of an excellent category with 19 nodes and 22 edges.

**Fig. 13:**
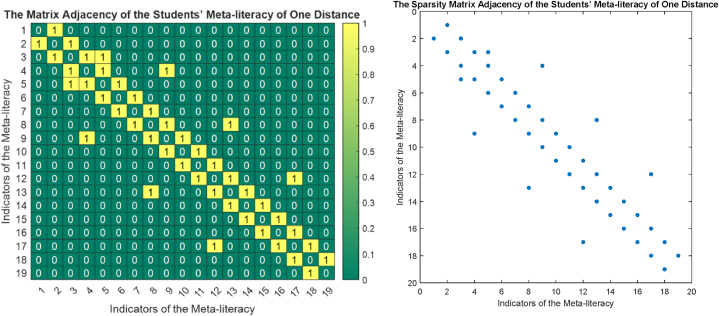
The matrix adjacency of the students' meta-literacy of one distance of student S20KE.

**Fig. 14:**
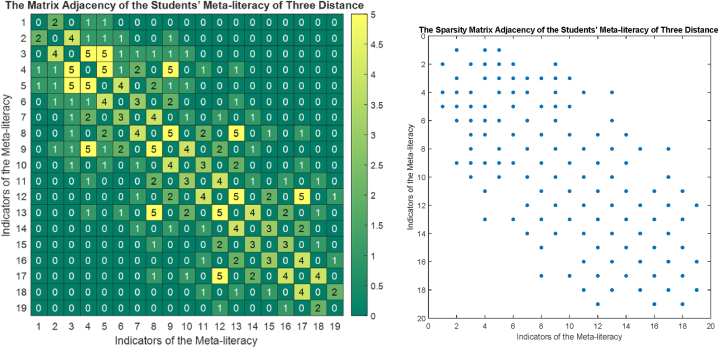
The matrix adjacency of the students' meta-literacy of three distances of student S20KE.

**Fig. 15:**
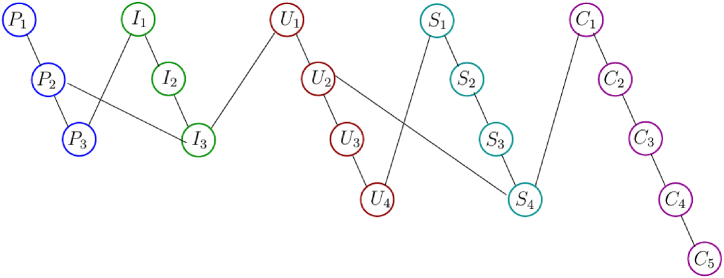
Phase portrait phase of meta-literacy of student S12KE of the good category with 19 nodes and 20 edges with good category.

**Fig. 16:**
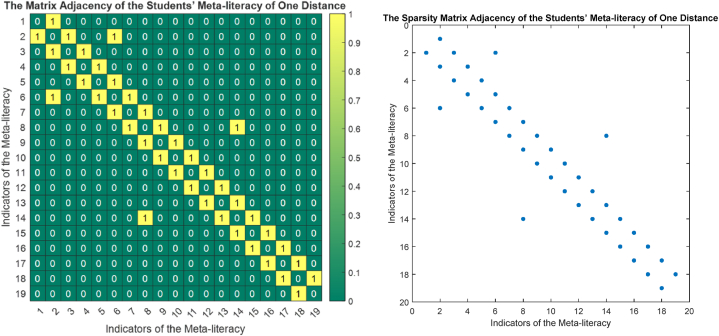
The matrix adjacency of the students' meta-literacy of one distance of student S12KE.

**Fig. 17:**
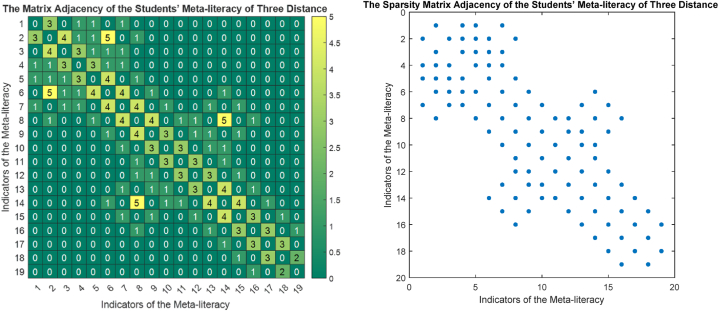
The matrix adjacency of the students' meta-literacy of three distances of student S12KE.

**Fig. 18:**
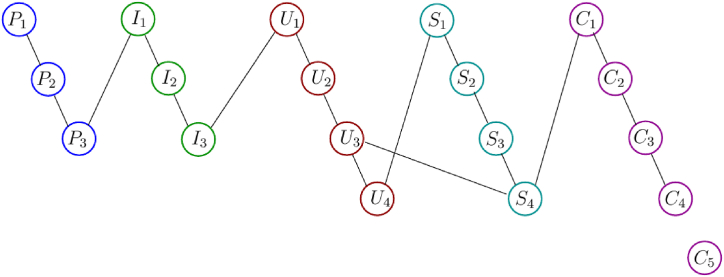
Phase portrait phase of the meta-literacy ability of student S8KE of the poor category with 19 nodes and 18 edges.

**Fig. 19:**
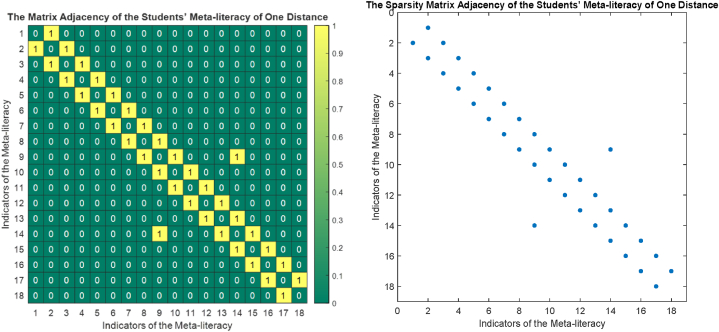
The matrix adjacency of the students' meta-literacy of one distance of student S8KE.

**Fig. 20:**
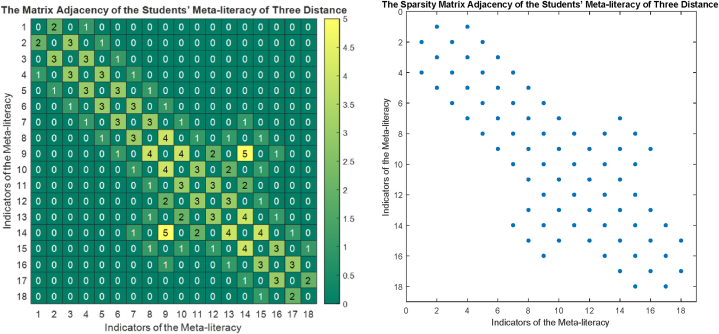
The matrix adjacency of the students' meta-literacy of three distances of student S8KE.

The phase portrait of the interview results above can be depicted in Fig. 12, Fig. 15, Fig. 18. Using MATLAB programming, this phase portrait can be analyzed in several ways: (1) Obtain the matrix adjacency of the students meta-literacy of one and three distance, then analyze the degree of each node, the number size of the phase portrait, the number of cycles and the number of walks of distance three. Fig. 13, Fig. 14, Fig. 16, Fig. 17, Fig. 19, Fig. 20 are successively presenting the matrix adjacency of the students' meta-literacy of one and three distance.

By the three phase portraits of the student meta-literacy, we can show that for the excellent category of S20KE research subjects, it gives that the meta-literacy ability is comprehensive, namely passing from P1 to C5. In general, the maximum degree of the matrix adjacency of distance one is greater than S20KE and S8KE, namely 4, 3, and 3. This shows that the meta-literacy process of S20KE research subject is more flexible than others since the meta-literacy flow shows many alternative paths in solving problems within the same duration of completion time. In contrast to the S8KE phase portrait, it shows the discontinuity from C4 to C5, which depicts that the meta-literacy groove is not reaching optimal. The maximum degree of the node of the S20KE, which achieve 4, is on node S3, and the number of edges is 24. While for S12KE and S8KE, the maximum degree of a node is 3, namely nodes S2, and S3, with the number of edges 19 and 18, respectively. The higher the number of edges suggests, the more complex their meta-literacy.

Lastly, the most important of the phase portrait analysis is the calculation of the integrity of the nodes [23]. It is stated that the integrity value can be determined from the calculation of RRA (Real Relative Asymmetry). The smallest RRA value indicates a high integrity value, meaning that the connectivity of the node with the low RRA in that phase portrait has the closest unity compared to the other nodes. Another meaning, the nodes with the smallest RRA indicate the most traversed and reached node during the process of meta-literacy.

The RRA score is calculated from the analysis of Total Depth (TD), mean Depth (MD), and Relative Asymmetry (RA) [24]. Total Depth (TD) is the total number of walk lengths of the observed sub-indicators, mean Depth $MD=\frac{TD}{n-1}$, $RA=\frac{2\left(MD-1\right)}{n-2}$, and $RRA=\frac{RA}{GL}$, where $GL=2\frac{n\sqrt{n}-2n+1}{\left(n-2\right)\left(n-1\right)}$. From this formula is obtained the distribution of values in Table 8.

**Table 8:** The Real Relative Asymmetry (RRA) distribution of student S20KE.

Nodes	J1	J2	J3	J4	…	J20	J21	J22	…	J44	J45	TD	MD	RA	GL	RRA
1	0	1	2	3	…	6	6	6				105	17.50	6.60	0.37	17.93
2	1	0	1	2	…	6	5	5				82	13.67	5.07		13.77
3	2	1	0	1	…	4	4	4				64	10.67	3.87		10.51
4	3	2	1	0	…	3	3	4	…	6	6	182	30.33	11.73		31.88
5	3	2	1	1	…	5	5	5				76	12.67	4.67		12.68
6	4	3	2	2	…	5	6					72	12.00	4.40		11.96
7	5	4	3	3	…	5	5	4	…			77	12.83	4.73		12.86
8	5	5	5	4	…	3	2	1	…			83	13.83	5.13		13.95
9	4	4	3	3	…	2	3	3	…			115	19.17	7.27		19.75
10	3	2	1	2	…	4	4	3	…			59	9.83	3.53		9.60
11	4	3	2	3	…	4	4	4	…			73	12.17	4.47		12.14
12	5	5	4	4	…	2	2	1	…			92	15.33	5.73		15.58
13	4	3	2	3	…	3	2	2				51	8.50	3.00		8.15
14	5	4	3	4	…	2	2	1				62	10.33	3.73		10.14
15	6	5	4	5	…	1	0	1	…			87	14.50	5.40		14.67
16	7	7	7	6	…	4	5	5	…			171	28.50	11.00		29.89
17	6	6	5	5	…	3	3	2	…			108	18.00	6.80		18.48
18	6	5	4	5	…	1	0	1				72	12.00	4.40		11.96
19	7	6	5	6	…	1	0					86	14.33	5.33		14.49

Table 8 shows the RRA distribution of each node of the phase portraits of research subjects S20KE, S12KE, and S8KE. It is obtained the fact that the lowest values of RRA are the nodes S3, S4, and U3 on the phase portraits of research subjects S20KE, S12KE, and S8KE, respectively. Thus, the three nodes have better integrity than the other nodes on each S20KE, S12KE, and S8KE. Table 9 summarizes the performance comparison according to the size, maximum degree, the number of walks of length three, and the minimum RRA.

**Table 9:** The phase portrait comparison of the students' meta-literacy of S20KE, S12KE, S8KE.

Student	Size	Max d(x)	Max A^3^	Min RA
**S20KE: Excellent meta-literacy**	25	4	5	8,15 (S3)
**S12KE: Good meta-literacy**	20	3	5	8,88 (S4)
**S8KE: Poor meta-literacy**	18	3	5	10,14 (U3)

From this comparison, it appears that the lowest RRA value is owned by students with excellent meta-literacy. It indicates that students with excellent meta-literacy ability have the strongest unity of the meta-literacy process among the meta-literacy nodes. Thus the meta-literacy sub-indicators are more thoroughly owned by them, starting from creating, incorporating, using, sharing, and collaborating.

## 4 Discussion

This study examines the implementation of RBL-STEM learning materials in improving students' meta-literacy ability to solve the problem of wallpaper tessellation decoration using local antimagic graph coloring techniques. We have been able to answer the two problems in this study. Firstly, how is the process of implementing the RBL model in the STEM approach in improving students' meta-literacy abilities in solving the problem of wallpaper decoration using local antimagic graph coloring techniques. We started the implementation by applying the RBL-STEM to the control class with no learning materials developed by researchers, whilst we applied the RBL-STEM together with learning materials developed by the researchers. The results show that there is a significant difference between the pre-test and post-test results, especially in the experimental class. The result is in line with the research that has been done by Hidayatul who focus in research implementation of research-based learning and the effect to the students' metacognition thinking skills in solving h-irregularity problem [19], and The implementation of RBL-STEM Learning Materials to Improve Students Historical Literacy [25]. The results showed that the descriptive data on student meta-literacy ability in the experimental class shows 10% of students have the poor meta-literacy ability, 17% of students have the fair meta-literacy ability, 26% of students have the good meta-literacy ability, 32% of students have the very good meta-literacy ability and 15% of students have the excellent meta-literacy ability. These results show a big improvement compared to the pre-test data.

Furthermore, we are also able to answer the problem of whether the implementation of the RBL model with STEM approach can improve students' meta-literacy ability in solving the problem of wallpaper decoration using graph coloring techniques or not. Based on the mean difference test, the *t*-test value was obtained on Sig. (2 Tailed) 0.000 < 0.05, this means that *t*_*count*_ >*t*_*table*_, which implies *H*_*0*_ is rejected and *H*_*1*_ is accepted. It concludes that there is a significant difference in meta-literacy ability between the control class and the experiment under the implementation of the RBL-STEM learning materials). The results of this study are in accordance with the research that has been done by Suntusia et al. [6]. They stated that the implementation of RBL is effective in improving the students' achievement in solving two-dimensional arithmetic sequence problems [6]. Another researchers who focus on the students' creative-innovative thinking skill in solving rainbow antimagic coloring under the implementation of the research-based learning model can be explored in Ref. [26]. Our research continued from the development of RBL-STEM learning instrument to improve the students' meta-literacy, see Ref. [22]. Futhermore [27], found that research-based learning is able to improve the students metacognition ability. In addition, the application of learning materials of RBL-STEM is much more effective than the application of other models in fostering the students higher order thinking skills [10].

## 5 Conclusion

Based on the research result, it is concluded that the implementation of the Research-Based Learning model with STEM approach has a significant influence on the students' metal-literacy ability to solve the wallpaper tessellation decoration problem using local antimagic graph coloring technique. However, during the RBL-STEM implementation in this research, to show the wallpaper printing process after the wallpaper tessellation decoration obtained using local antimagic graph coloring, we had some difficulties. During the implementation, we used glossy photo paper to print it. Actually, students need to be shown how to print on real wallpaper paper and how to stick it on a house building. Therefore, further research is needed by applying project-based learning with the STEM approach in developing students' creative skills by sticking wallpaper on the house-building prototype. Thus, the process of developing a house-building prototype is also challenging for the next research.

## Author contribution statement

Dafik: Conceived and designed the experiments; Performed the experiments; Analyzed and interpreted the data; Contributed reagents, materials, analysis tools or data; Wrote the paper.

Kadir; Zainur Rasyid Ridlo: Conceived and designed the experiments; Performed the experiments; Contributed reagents, materials, analysis tools or data; Wrote the paper.

T K Maryati: Performed the experiments; Analyzed and interpreted the data; Contributed reagents, materials, analysis tools or data; Wrote the paper.

Sufirman: Conceived and designed the experiments; Performed the experiments; Analyzed and interpreted the data; Wrote the paper.

## Data availability statement

Data included in article/supp. material/referenced in article.

## Additional information

No additional information is available for this paper.

## Declaration of competing interest

The authors declare that they have no known competing financial interests or personal relationships that could have appeared to influence the work reported in this paper.
